# Systematic Review of Managed Care Medicaid Outcomes Versus Fee-for-Service Medicaid Outcomes for Youth in Foster Care

**DOI:** 10.1007/s11414-025-09987-7

**Published:** 2026-01-13

**Authors:** Emma R. Breen, Nosherwan Yasin, Zhidi Luo, Annie B. Wescott, Richard A. Epstein

**Affiliations:** 1 Department of Psychiatry and Behavioral Sciences, Northwestern University Feinberg School of Medicine, Mental Health Services and Policy Program, 710 North Lake Shore Drive, Chicago, IL 60611 USA; 2 Galter Health Sciences Library & Learning Center, Northwestern University, Chicago, IL 60611 USA

## Abstract

Transitioning foster care children from fee-for-service (FFS) Medicaid to Medicaid managed care (MMC) plans is increasingly common, yet research on the impact of such transitions is limited. This systematic review addresses this gap by synthesizing evidence on the effects of transitioning foster care children from FFS to MMC on healthcare utilization and costs. Eligibility criteria included peer-reviewed articles on youth in foster care in the United States, aged 0–18 years, comparing selected outcomes under FFS vs. MMC. The outcomes of interest included behavioral, dental, vision, and well-child care. The databases searched were MEDLINE (PubMed), Cochrane Library (Wiley), APA PsycINFO (Ebsco), Social Services Abstracts (ProQuest), and Web of Science (Clarivate). A qualitative synthesis of articles meeting the inclusion criteria was performed. Five articles met the inclusion criteria. Three articles yielded mixed findings regarding behavioral healthcare, which was evaluated as having low certainty of evidence. Two articles on well-child visits indicate significant changes when transitioning youth from FFS to MMC coverage, with varied impacts based on how well-child visits were defined and rated moderate certainty of evidence. No articles examined dental or vision outcomes. Results suggest a shortage of empirical evidence on the effects of transitioning from FFS to MMC for children in foster care. Future research should describe insurance benefits packages in greater detail, as not all FFS or MMC programs are the same and continue to study the impacts of such transitions on healthcare utilization and outcomes for this vulnerable group.

## Introduction

Nearly all children in the foster care system in the United States have access to Medicaid services through Title IV-E of the Social Security Act.^1^ This provision ensures that youth in the foster care system, who are known to be at increased risk for many health and behavioral health conditions,^2^ have access to comprehensive health, behavioral health, dental, and vision services. Ensuring access to comprehensive healthcare is essential for ensuring that youth receive the assistance necessary for their well-being and development.

Most states administer Medicaid services to children in foster care through traditional fee-for-service (FFS) Medicaid plans. However, states are increasingly opting to transition children in foster care to Medicaid managed care (MMC) plans.^3^ Proponents of MMC argue that it will enhance healthcare availability, accessibility, and affordability.^4^ Research on MMC in the general Medicaid population has yet to reach a consensus on whether managed care positively or negatively affects access and care quality, with outcomes varying by plan design, provider network adequacy, and care‑management strategies.^5^ It remains unclear which of these findings are applicable to foster care youth, who face unique challenges such as placement instability, fragmented care coordination, and complex behavioral health needs. These factors may influence how foster youth experience transitions in healthcare delivery systems, making it essential to study this population separately.^6^

Existing literature on the effects of transitioning to MMC in other populations provides limited insight into how such changes affect foster youth. This systematic review summarizes existing empirical evidence about the impacts of transitioning children in foster care from FFS to MMC plans, focusing on healthcare utilization and cost outcomes. To help guide future policy and practice decisions, there is an urgent need to determine the comparative effectiveness of FFS and MMC plans in meeting foster care children’s needs.^6,7^ By examining studies specific to foster youth, this review aims to clarify whether the impacts of MMC differ from those observed in other populations and to identify areas where further research is needed.

## Methods

The study protocol was registered with PROSPERO in September 2023 and is accessible to the public under ID number CRD42023461338. The study team, which includes a research librarian, developed a comprehensive existing literature search strategy (Appendix). The search used a combination of controlled vocabulary and title/abstract terms related to youth in foster care with MMC versus FFS programs. Database-specific syntax and language were adapted for each database, and the search was executed on a single day without the use of additional filters or limits. Literature was collected from various sources, including MEDLINE (PubMed), Cochrane Library (Wiley), APA PsycINFO (Ebsco), Social Services Abstracts (ProQuest), and Web of Science (Clarivate). The inclusion criteria required that the study population be youth in foster care, aged 0 to 18 years. No limits were applied to the timeframe or to the date of publication.

All results were imported into citation management software (EndNote) for deduplication and uploaded to a screening platform (Rayyan). Two reviewers independently assessed titles and abstracts for eligibility, resolving discrepancies through group discussion and consensus among the screeners. Final eligibility decisions were made following independent full-text reviews by the same two authors who served as the screeners. A third author resolved disagreements regarding eligibility.

Included articles needed to be peer-reviewed, compare FFS to MMC, and exclude gray literature. Only articles in the United States were considered, as the research question pertains specifically to the US healthcare system. Key data elements from included articles were extracted using a structured form in Excel, focusing on specific healthcare utilization and cost outcomes. Utilization or cost outcomes were categorized under the specific healthcare domains of behavioral healthcare, dental care, vision care, and well-child visits.

A qualitative synthesis of results was conducted, with relevant information extracted and categorized separately for each outcome. There were not enough included articles to conduct a meta-analysis. Reporting bias was calculated using risk of bias in nonrandomized studies of interventions (ROBINS-I).^8^ GRADE, or Grading of Recommendations, Assessment, Development, and Evaluations, was used to assess the certainty of evidence.^9^

## Results

**Figure 1:**
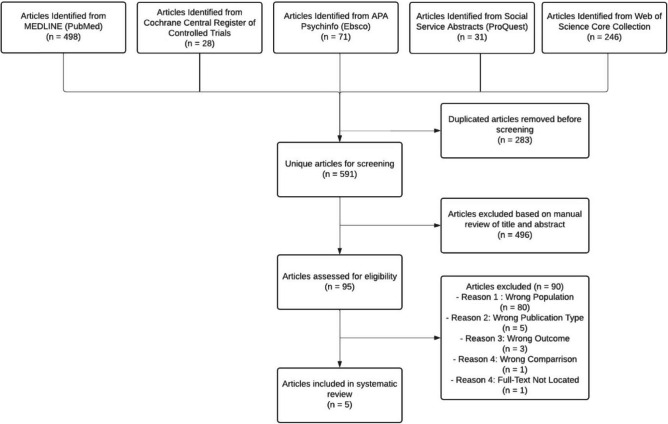
Flow diagram

The literature search resulted in 498 articles from MEDLINE, 28 articles from Cochrane Central Register of Controlled Trials, 71 articles from APA PsycINFO, 31 articles from ProQuest, and 264 articles from Web of Science Core Collection. Then, 283 articles were duplicates, resulting in 591 unique articles for screening titles and abstracts. After screening titles and abstracts, 496 articles were excluded, leaving 95 articles for full-text review. Then, 80 of the 95 articles were excluded for studying the wrong population, five were excluded for being the wrong publication type, three were excluded for looking at the wrong outcome, one article was excluded for having the wrong comparison group, and one article’s full text could not be obtained (Fig. 1).

After the full-text review, five articles met the eligibility criteria. Three articles examined the impact on youth in foster care of a transition from FFS to MMC on behavioral healthcare utilization. One of these three articles also explored behavioral healthcare cost. The two remaining articles explored the utilization of well-child services.

Several articles displayed high to moderate risks in various domains like confounding, selection of participants, and missing data (Table 1). Differences in billing practices across states (e.g., use of UB-04 vs. 1500 forms for residential treatment services) may affect the comparability of utilization measures across studies. The included articles lack certain specifics, such as information about how Federal Qualified Health Center (FQHC) visits or T1015 claims were counted within the behavioral healthcare articles. Another example is that age inclusion criteria varied slightly across studies. While most focused on youth aged 0–18, some included individuals up to age 21, reflecting differences in state Medicaid eligibility for foster youth. These variations are noted in the data extraction table (Table 2).

**Table 1:** Risk of bias (ROBINS-I)

	*Confounding*	*Selection of participants*	*Classification of interventions*	*Deviations from intervention*		*Missing data*	*Measurement outcomes*
*Cuellar, 2001*	High	Moderate	Low	Moderate	Moderate		High
*Libby, 2002*	Moderate	High	Low	High	Moderate		High
*Snowden, 2003*	Moderate	Low	Low	Low	High		Low
*Bright, 2018*	High	Moderate	High	Low	Moderate		Moderate
*Day, 2016*	Moderate	Moderate	Low	Low	Moderate		Low

**Table 2:** Summary of findings

	*FSS*	*MCM*	*Relative effect*	*No. of participants*			*GRADE*	*Comments*			
*Behavioral healthcare utilization*	The FFS group saw higher inpatient and RTC utilization, while outpatient use decreased but the number of visits among users increased. The fee-for-service group showed decreases in service utilization, but these changes were not necessarily statistically significant	The FFS group had more inpatient and RTC utilization, with fewer outpatient visits, but more visits per user. The fee-for-service group had decreased service utilization, but the changes were not necessarily statistically significant	The introduction of managed care led to reduced youth inpatient services and lower outpatient use in not-for-profit settings compared to fee-for-service settings. Managed care coincided with decreases in predicted average inpatient days for White, Black, and Hispanic children, with the most pronounced decline observed for White children. Managed care did not significantly impact outpatient use relative to fee-for-service	9010		Low			The included literature regarding behavioral healthcare utilization is rated with low certainty of evidence due to its high risk of bias and indirectness		
*Behavioral healthcare cost*	Regression coefficients do not offer postperiod data exclusive to the FFS comparison group	Inpatient and residential treatment costs decreased, while outpatient costs increased. There was also observed cost shifting towards services covered by child welfare budgets compared to health insurance	Pre–poststudy design for expenditure data	4030		Low		The included literature regarding behavioral healthcare cost is rated with low certainty of evidence due to displaying a high risk of bias and indirectness			
*Dental uilization*			No eligible literature								
*Dental cost*			No eligible literature								
*Vision utilization*			No eligible literature								
*Vision cost*			No eligible literature								
*Well-child care utilization*	Access to primary and preventative care for the FFS group remained relatively stable or slightly decreased from 2006 to 2010. The proportion of foster youth classified as ED-reliant decreased across all age groups within the FFS group. However, this study did not compare the FFS group to any other outside of the policy change context in Michigan	MC was associated with improved access to primary and preventive care, especially for older youth. Transition to MC was linked to improved timeliness of well-child visits for older youth in care	Findings suggest that managed care (MC) may improve access to primary care for certain age groups and promote preventative care utilization among foster youth. Additionally, it was found that the MC group in Texas and the fee-for-service (FFS) group in Florida both experienced decreases in emergency department reliance after the transition	64,837		Low		Well-child care utilization findings are given a low certainty of evidence. One of the two relevant articles has a high risk of bias, and the other has a low risk of bias. The number of studies contributes to the low score			
*Well-child care cost*			No eligible literature								

Moreover, while Medicaid claims and encounter data, along with specific procedure and diagnosis codes, are commonly used for service definitions and counting, the comparability of utilization and cost outcomes across multiple states is not consistently addressed or fully supported by the sources. For instance, Bright, Kleinman, Vogel, et al. (2018) attempt a multistate comparison but note limitations, while the other studies are single-state focused and highlight state-specific policy and billing practice variations that could hinder direct cross-state comparisons.

Table 2 summarizes findings on healthcare utilization and costs. This table displays that managed care (MC) reduced inpatient services and improved access to primary care, while fee-for-service (FFS) had higher inpatient and residential treatment center utilization. The certainty of evidence varies, with behavioral healthcare findings rated low due to high bias, and well-child care utilization rated moderate (Table 2).

### Behavioral healthcare

Three included articles examined behavioral healthcare outcomes. These articles all drew from the same dataset originating from the Colorado Capitation Pilot Program.^10–12^ To prevent redundancy, a general overview of the Colorado Capitation Pilot Program is provided, followed by a breakdown of the methodologies and findings presented in each related article. The results were then synthesized into utilization outcomes and cost outcomes. All of the articles contain data on behavioral healthcare utilization,^10–12^ and one reported cost savings data.^11^

The Colorado Medicaid Capitation Pilot Program, initiated in 1995, aimed to reform mental health care delivery by transitioning from FFS to an MMC model. This involved implementing a capitated managed care system in previously FFS mental health treatment facilities. The state partnered with six not-for-profit community health centers to serve as the managed care organization (MCO), each responsible for a specific geographic area. The state also contracted with three joint ventures between a for-profit managed care company and several community mental health centers designated as "”for-profits.” Additionally, three mental health centers operating under the existing FFS system also participated in the pilot program by serving as the comparison group.

This pilot program aimed to assess the impact of the capitated system on behavioral healthcare utilization compared to facilities operating under the traditional FFS model, not limited to the child welfare population. Previous articles on this pilot program demonstrated that both capitated MMC models increased outpatient utilization, which includes individual and group therapy, crisis and evaluative services, case management, and day treatment programs, measured per service.^10–12^ Conversely, the cost of inpatient care, which is defined by days in a psychiatric hospital stay or acute, general hospital days identified with a mental health diagnosis, decreased among adults with serious mental illness, compared to FFS.^13^ Similar cost savings were observed for youth under the age of 18. However, findings regarding utilization were mixed, with more youth accessing services from for-profit MMC sites than non-profit MMC sites or FFS sites.^14^ Following these initial articles, researchers analyzed outcomes specific to Colorado’s child welfare population, including the three articles that met the inclusion criteria for this systematic review.

Cuellar, Libby, and Snowden (2001)^10^ conducted a study to examine mental health service utilization rates specific to Colorado youth involved in the juvenile justice or child welfare systems. This review includes only findings from the article pertaining to the child welfare sample. The study utilized a quasi-experimental, pre–post design with nonequivalent comparison groups, employing a two-part model to analyze service patterns. Dependent variables included the probability of service use and the number of services used (days or visits), with demographic variables and provider types as explanatory variables. Cuellar, Libby, and Snowden’s article (2001) was rated as having a high risk of bias due to confounding variables such as non-random assignment and local variability. Additionally, outcome measurement issues with user sample denominators and the exclusion of services typically covered by managed care lead to potential underestimation of costs and incomplete utilization results. Additionally, managed care plan enrollment was not randomly assigned.

Data from 1994 to 1997 was analyzed across a pre-program implementation phase and two post-implementation phases. The data was gathered from two experimental MC sites (one not-for-profit and one for-profit) and one comparison FFS site. The data was obtained through Medicaid claims, a “shadow billing” system for MCOs, and child welfare encounter data.

The child welfare sample was comprised of 9010 Colorado Child-Welfare involved youth under 18 years old, including those with in-home or out-of-home placements.^10^ Among the 9,010 young people, 59% were male in the pre-period, 58% in postperiod 1, and 60% in postperiod 2.^10^

The authors observed a decrease in the probability of inpatient service utilization, measured by days, in both not-for-profit (17%, 10%, and 10%) and for-profit managed care sites (22%, 15%, 15%) across the pre- and postimplementation periods.^10^ At the FFS comparison site, the probability of inpatient service use increased from 14% during the pre-period to 21% during postperiod 1 and 19% during postperiod 2.^10^ The number of inpatient days remained relatively stable across sites.^10^

The probability of utilizing outpatient services decreased in both not-for-profits and for-profit managed care sites across different study periods.^10^ Specifically, at not-for-profits, the probabilities were 90%, 80%, and 85% during the preperiod, postperiod 1, and postperiod 2, respectively, while for-profit sites showed probabilities of 91%, 87%, and 90% during the same periods.^10^ At the FFS comparison site, the probability of using outpatient services decreased from the pre-period to post-period 1 (81% to 78%) but returned to the pre-period rate by post-period 2.^10^

The number of individual outpatient services during a course of treatment increased significantly across all sites.^10^ Notably, not-for-profit managed care sites delivered an average of 16.5 services per user during the preperiod, 24 during postperiod 1, and 31.6 during postperiod 2.^10^ For-profits provided an average of 13.3 services per user during the preperiod, then 19.4 and 25.6 during the respective postperiods.^10^ The FFS site delivered an average of 13.3 services, then 19.3 and 25.4, respectively.^10^

Conversely, the likelihood of using RTCs (residential treatment centers) increased in both nonprofit and for-profit sites over the study periods.^10^ In nonprofits, the likelihood rose from 11% to 30% to 33%, while in for-profits, it increased from 12% to 21% to 24%.^10^ Comparatively, at FFS sites, the probability of use increased from 11% to 17% to 22%.^10^ Only the FFS site exhibited statistically significant changes in average days in treatment (62.6, 51.6, and 74.0, pre- and post-periods, respectively).^10^

Libby, Cuellar, Snowden, et al. (2002)^11^ examined shifts in service expenditures during the Colorado Capitation Pilot Program. Data was collected from a pre-program implementation phase and two post-implementation phases. The information was sourced through Medicaid claims, a “shadow billing” system used by managed care organizations, and data related to encounters within the child welfare system. Libby, Cuellar, Snowden, et al.’s article (2002) was also rated as having a high risk of bias, particularly in participant selection and intervention deviations, with contracts awarded based on proposals rather than random assignment. The article also highlighted cost-saving behavior due to the shift to child-welfare-covered services instead of focusing strictly on MMC services (Table 1).

The total sample consisted of 48,403 youths under the age of 19, with Medicaid claims data from October 1994 to June 1997.^11^ There were 3,931 child welfare–involved youth in the preperiod, 3532 in postperiod 1, and 4030 in postperiod 2.^11^ Service utilization and cost changes were analyzed by service type: inpatient, outpatient, and therapeutic residential treatment. Findings are represented by a regression coefficient of service use and service expenditures using a “difference in differences” methodology.

Findings regarding service utilization for the child welfare sample reveal a significant increase in inpatient and residential treatment use (81% and 42%, respectively), and outpatient use fell by 47% compared to the FFS control group.^11^ Findings regarding expenditures exclusively related to the child welfare sample reveal that, compared to the FFS control group, inpatient and residential treatment costs decreased significantly (23% and 65%, respectively), and outpatient costs increased (9%).^11^

The article’s broader conclusions, extending beyond just child welfare cases, reveal a significant trend: expenses are increasingly being redirected to services funded by child welfare budgets, rather than those covered by health insurance plans.^11^ Total expenditures decreased by 16%, while residential costs increased by 13%.^11^ These findings suggest expenditure patterns indicative of cost substitutions for services not covered by MMC.^11^

Snowden, Cuellar, and Libby (2003)^12^ examined if there are varying effects of transitioning youth in foster care to MMC by race. The study used a quasi-experimental, pre-post design involving a "difference-in-difference" analysis with nonequivalent comparison groups. It explored the shift of public mental health providers from a FFS to a capitated MMC system. The study period was divided into three time periods, reflecting one pre-implementation period and two post-periods. Snowden, Cuellar, and Libby^12^ examined both “access,” referring to the probability of being referred to services and “utilization,” pertaining to the amount of care received once referred. Snowden, Cuellar, and Libby (2003) was rated as having a moderate risk of bias due to lower risk in participant selection compared to other Colorado Pilot program articles, but still containing the potential confounding factors of nonrandom assignment and local variability, and possible missing data from underreporting in shadow billing datasets.

The sample of 60,324 youth was based on all state child welfare Medicaid claims data from September 1994 to June 1997.^12^ The three subsamples analyzed were White/non-Hispanic youth (*n* = 36,360), Black youth (*n* = 8032), and Hispanic youth of any race (*n* = 17,932). Then, 52% of the study population was male, and the largest age group was 5 years old (38%).^12^

The analysis revealed that MMC did not have a statistically significant effect by the second post-period on access for White youth in comparison to FFS.^12^ Black youth initially experienced increased access to care in the first post-period (23%), but this increase became statistically insignificant by the end of the second post-period.^12^ Hispanic youth did not experience a significant change in access during the first post-period; however, by the end of the second post-period, they exhibited a 22% increase in the probability of receiving services.^12^

Subsequently, changes in service utilization by service type were measured. Service types were defined as days in inpatient care, number of outpatient visits, and days in residential treatment. By the end of the study period, Black and White youth were observed to have fewer days in inpatient care under MMC than FFS, and there was no difference in days for Hispanic youth.^12^ The number of outpatient visits remained comparable between MMC and FSS.^12^ By the end of the study period, White youth were receiving an average of 3.6 visits under FFS and 3.9 under MMC, Black youth were receiving 3.2 visits under FFS and 3.4 under MMC, and Hispanic youth received slightly fewer with FFS (2.1 visits) than with MMC (2.8 visits).^12^

All youth received fewer days in residential treatment under MMC than FFS, with the predicted average days in residential treatment for white youth being 9.5 under FFS sites and 7.4 under MMC.^12^ For Black youth, the predicted average was 5.9 days and 4.9 days, FFS and MMC, respectively.^12^ For Hispanic youth in care, the predicted average was 5.9 days and 4.6 days.^12^

Using the GRADE approach, the literature regarding behavioral healthcare utilization is rated with low certainty of evidence. This low rating is attributed to the high risk of bias in the articles and their indirectness. There are concerns about the reliability and applicability of the findings related to how managed care affects the use of behavioral healthcare services, including inpatient, residential treatment, and outpatient care. The certainty of evidence for behavioral healthcare costs is also rated as low for displaying a high risk of bias and indirectness. This indicates that there is limited confidence in the accuracy of the reported cost changes associated with managed care.

### Well-child visits

Bright, Kleinman, Vogel, et al. (2018)^15^ investigated the variations in primary care, preventive care, and emergency department (ED) usage among foster youth in Texas (*n* = 38,569) who switched to the specialty managed care plan, STAR Health, from FFS, compared to foster youth in Florida (*n* = 24,611) who remained on FFS from 2006 to 2010.^15^ It is notable that STAR Health contains features that encourage coordinated care and provider acceptance, such as a 24-h nurse hotline. The article focused on foster youth aged 0 to 18 actively involved in cases with Title IV-E eligibility and enrolled in either Texas or Florida Medicaid during the study period. Only data on primary care and preventive care are included in this review. Bright, Kleinman, Vogel, et al.’s article (2018) was rated as having a high risk of bias due to biased participant selection, excluding youth with shorter stays in care, and unaddressed confounding variables from concurrent interventions.

Well-child visits encompass both primary and preventive care, serving the dual purpose of addressing both the immediate health needs of the child and preventing potential health issues through regular check-ups, screenings, and vaccinations. Access to primary and preventive care was assessed using age-stratified groups with continuous enrollment. For primary care, individuals needed at least one visit with a primary care provider (PCP) during the measurement year (or the previous year, depending on age). For preventive care, age-stratified groups were evaluated for well-child visits indicated by procedure or diagnosis codes with a PCP or obstetrician/gynecologist during the calendar year.

Over the study period, access to primary and preventative care improved for youth in foster care covered by MMC, unlike youth in the FFS control group.^15^ For foster youth aged 12 to 24 months, there was no significant change in access to primary care after transitioning to MMC.^15^ For youth aged 2 to 6 years, transitioning to MMC improved access to primary care by 12.18%, 13.01% for youth aged 7 to 11 years, and 8.68% for youth aged 12 to 18 years after adjusting for covariates.^15^ Similarly, preventive care access improved by 13.81% and 10.47% for youth aged 3 to 6 years and 12 to 18 years, respectively.^15^

Day, Curtis, Paul, et al. (2018)^16^ examined the timeliness of well-child visits for older foster care youth, 10 to 20 years old. Data was captured from November 1, 2009, to September 1, 2012, during which the state of Michigan transitioned its youth in care from FFS to Health Maintenance Organization (HMO) plans, a type of MMC. A retrospective analysis was conducted for 1,657 youth using child welfare administrative data in addition to Medicaid claims and encounter data.^16^ The timeliness of well-child visits was determined by analyzing the initial well-child visit recorded following entry into foster care, serving as the reference point. The duration between foster care entry and the first well-child visit was calculated, and visits occurring within 30 days of entry were considered timely. This study coincides with the introduction of policies designed to encourage timely Medicaid enrollment and well-child visits for youth newly entering care, with confounding factors addressed in the analysis. Day, Curtis, Paul, et al.'s article (2016) was rated as having a low risk of bias.

The percentage of timely visits increased during the managed care period, from 27.6% during the FFS period to 52.2% during the MC period.^16^ Additionally, the percentage of youth meeting the requirement to begin Medicaid within 14 days of foster care entry rose from 85.1% to 96.8% during the FFS and MMC (HMO) periods, respectively.^16^ The reported odds ratio for receiving timely well-child visits reveals that youth who entered care during the MMC (HMO) period were 2.46 times as likely as those who entered care during the FFS period.^16^

Notably, this analysis revealed that non-Hispanic black foster care youth and those with multiple housing arrangements had lower odds (0.65) of timely visits.^16^ Similarly, four (0.78) or more (0.62) housing placements and those not enrolled in Medicaid within 14 days of foster care entry (0.46) had lower odds of timely visits.^16^ Regarding the time taken for the first well-child visit, youth entering foster care during the managed care period experienced significantly fewer days to their first visit compared to those in the FFS or transition periods. Overall, the analysis indicated that the transition to managed care was associated with improved timeliness of well-child visits among foster care youth, with notable disparities observed based on demographic and enrollment factors.

Well-child visit utilization is rated with a low certainty of evidence, primarily due to the limited number of eligible articles, only two, and concerns about risk of bias. One study was found to have a high risk of bias across several domains, while the other was assessed with a low risk of bias. The small evidence base and methodological limitations together weaken the overall confidence in the findings.

### Dental and vision care

No included articles assessed the impact of MMC on dental or vision care utilization or cost for foster youth.

## Discussion

Given that states are increasingly considering whether to provide Medicaid services to youth in the foster care system using FFS or MMC programs, it is critically important to understand what we already know about the impact of these different health insurance models on healthcare utilization and cost for this important population of vulnerable youth. This review suggests there is relatively little existing literature on the topic.

Regarding behavioral healthcare utilization and cost, articles on the Colorado Capitation Pilot Program offer insight into changes in utilization caused by shifting from FFS to MMC. There was an observed decrease in the likelihood of being referred to inpatient care, and no significant change in the number of days spent in inpatient care if referred.^10–12^ There was a decrease in outpatient care; however, the number of outpatient sessions, if referred, increased.^10–12^ The likelihood of being referred to RTC increased, but not the number of days spent in RTC, when looking at MC sites.^10–12^ It is notable that, regardless of the insurance model, Hispanic youth are less likely to receive care than their White counterparts.^12^ Similarly, Black youth did not experience service use changes specific to MC. Black youth received more care than Hispanic youth but less care than White youth.^12^

There is limited data to suggest that MC affects behavioral healthcare expenditures for youth in foster care. One study observed that costs fell for inpatient and residential but rose for outpatient.^11^ Of the total study sample, not exclusive to youth in care, cost-shifting behavior is observed away from services covered by managed care to services covered by child welfare agency budgets.^8^ Namely, expenditures shift from inpatient care to residential treatment.^11^

Two articles examined the differences in well-child visits (healthcare access and utilization) among foster youth during transitions from FFS to MMC systems. One study examined variations in primary/preventive care and emergency department (ED) usage among foster youth in Texas and Florida transitioning to MC from FFS Medicaid. The study found that transitioning to MC was associated with improved access to primary and preventive care, especially for older youth.^15^ The speculation centered on the alignment of MC features, such as provider networks and care coordination, with the complex needs of foster youth. Another study, focused on the timeliness of well-child visits for older foster youth, found that the transition to MC was linked to improved timeliness, with significant disparities based on demographic and enrollment factors.^16^

## Conclusion

The systematic review reveals significant changes in the child welfare population as they transition from Fee-For-Service (FFS) to Medicaid Managed Care (MMC) models, with the impact varying by service type. Findings on broader Medicaid populations, while mixed, suggest potential benefits that may have encouraged this shift to MMC across many states. However, the effectiveness of this transition in improving healthcare outcomes for youth in care remains unclear due to mixed evidence and limited research.

This review emphasizes the urgent need for further empirical investigation to uncover the outcomes associated with MMC adoption within child welfare systems. Comprehensive data on usage patterns, cost implications, and overall healthcare quality are crucial to inform evidence-based decision-making and policy development in this area. Future research efforts should prioritize acquiring thorough data to provide a more definitive understanding of the comparative effectiveness of healthcare models for youth in care.

## Implications for Behavioral Health

The studied transitions from Fee-For-Service to Managed Care were associated with changes in the utilization of behavioral healthcare services. The likelihood of being referred to inpatient and outpatient care decreased under MMC compared to FFS.^10–12^ While the number of days spent in an inpatient setting or residential care remained similar, the number of outpatient services received, if referred, increased substantially in both MMC and FFS sites.^10–12^ The differential impact on access and utilization among racial groups stresses the importance of addressing disparities in behavioral healthcare to ensure equitable service delivery across diverse populations.^12^

The MMC model also achieved cost savings, particularly in reducing inpatient care costs, but this was accompanied by an increase in residential treatment costs, which, in this case, were covered by child welfare budgets.^11^ Observing cost-shifting behavior reveals the need to accurately capture cost-saving measures and achieve comprehensive service provision.

It is important to note that individuals may perceive outcomes as positive or negative based on their clinical perspective. “More care” may not always be positive if the form of care is less preferred than others. For example, some may regard the finding that the likelihood of receiving residential care increases under MMC as negative if they have a negative perception of residential treatment.^17^ Additionally, some individuals may want to reduce the probability of being referred to inpatient treatment, but only if it means a greater likelihood of being referred to outpatient services, which does not currently appear to be the case.^10–12^ The overall lack of empirical evidence, however, has the most significant implications for behavioral healthcare, by highlighting the need for more robust evidence to guide decision-making about how to best provide Medicaid services to youth in the foster care system.

## Limitations

This systematic review includes a notably limited number of articles. Three of the five articles were derived from the same parent study, further restricting the generalizability of the results. Additionally, the articles exhibit a high risk of bias due to confounding factors, participant selection, and missing data. The heterogeneity among the included articles, including varying methodologies, populations, and outcome measures, complicates the synthesis of results. It is worth noting that large-scale societal factors, such as the Affordable Care Act, as well as other policies and events, may limit the generalizability of these studies and should be taken into consideration. Lastly, the lack of standardized definitions for key outcomes, such as well-child visits and behavioral healthcare utilization in the underlying literature, makes comparisons across articles require caution.

## Data Availability

The data extracted from the included articles and any other materials used in the review are available upon request.

## Appendix

Full Search Strategy

**Table:**

Database searched	Date searched	Results
MEDLINE (PubMed)	9/13/2023	498
Cochrane Central Register of Controlled Trials Issue 8 of 12, August 2023	9/13/2023	28
APA Psycinfo (Ebsco)	9/13/2023	71
Social Services Abstracts (ProQuest)	9/13/2023	31
Web of Science Core Collection (Clarivate)	9/13/2023	246
Total		874
After de-duplication		591

### Example search methods

A comprehensive search of the literature was developed in partnership with a research librarian (ABW). The search strategy combined database-specific controlled vocabulary and title/abstract terms related to (1) children in care, (2) Medicaid managed care, and (3) fee-for-service plans. An initial MEDLINE (PubMed) search was adapted to the following databases: Cochrane Central Register of Controlled Trials (CENTRAL), APA Psycinfo (Ebsco), Social Services Abstracts (ProQuest), and Web of Science (Clarivate). All databases were searched from inception to present without filters or limits. Results were downloaded and underwent multipass-deduplication in a citation management software (EndNote). Unique records were then uploaded to a screening platform for title/abstract screening by two independent reviewers.

**Table:**

MEDLINE (PubMed)		
Number	Search	Results
1	(“Child”[Mesh] OR “Child, Orphaned”[Mesh] OR “Child, Foster”[Mesh] OR “Adolescent”[Mesh] OR “Pediatrics”[Mesh] OR “Foster Home Care”[Mesh] OR child[tiab] OR children*[tiab] OR childhood[tiab] OR kid[tiab] OR kids[tiab] OR adolescent[tiab] OR adolescents[tiab] OR adolescence[tiab] OR preteen*[tiab] OR teenager*[tiab] OR teen[tiab] OR teens[tiab] OR toddler[tiab] OR toddlers[tiab] OR preschool*[tiab] OR school-age*[tiab] OR girl[tiab] OR girls[tiab] OR girlhood[tiab] OR boy[tiab] OR boys[tiab] OR boyhood[tiab] OR youth[tiab] OR pediatric[tiab] OR pediatrics[tiab] OR Foster-care[tiab] OR foster-home[tiab] OR state-care[tiab] OR foster-parent*[tiab] OR foster-mother*[tiab] OR foster-father*[tiab] OR kinship-care[tiab] OR kinship-provider*[tiab] OR guardian[tiab] OR guardians[tiab] OR guardianship[tiab] OR DCSF[tiab]) AND (“Managed Care Programs"[Mesh] OR "Medicaid"[Mesh] OR Managed-care[tiab] OR Medicaid[tiab] OR MMC[tiab] OR MCO[tiab] OR coordinated-care[tiab] OR coordinated-health-care[tiab] OR HMO[tiab] OR health-maintenance-organization*[tiab] OR PPO[tiab] OR Preferred-Provider-Organizations[tiab] OR POS[tiab] OR point-of-service-plan*[tiab] OR EPO[tiab] OR exclusive-provider-organization*[tiab]) AND (“Fee-for-Service Plans”[Mesh] OR Fee-for-Service[tiab] OR fee-for-services[tiab] OR fees-for-service[tiab] OR fees-for-services[tiab] OR FFS[tiab])	498

**Table:**

Cochrane Central Register of controlled trials Issue 8 of 12, August 2023		
Number	Search	Results
#1	MeSH descriptor: [child]	78,477
#2	MeSH descriptor: [child, orphaned]	46
#3	MeSH descriptor: [child, foster]	13
#4	MeSH descriptor: [adolescent]	125,806
#5	MeSH descriptor: [pediatrics]	1179
#6	MeSH descriptor: [foster home care]	180
#7	(child OR children* OR childhood OR kid OR kids OR adolescent OR adolescents OR adolescence OR preteen* OR teenager* OR teen OR teens OR toddler OR toddlers OR preschool* OR school-age* OR girl OR girls OR girlhood OR boy OR boys OR boyhood OR youth OR pediatric OR pediatrics OR Foster-care OR foster-home OR state-care OR foster-parent* OR foster-mother* OR foster-father* OR kinship-care OR kinship-provider* OR guardian OR guardians OR guardianship OR DCSF):ti,ab,kw	300,462
#8	#1 OR #2 OR #3 OR #4 OR #5 OR #6 OR #7	300,530
#9	MeSH descriptor: [managed care programs]	590
#10	MeSH descriptor: [Medicaid]	387
#11	(Managed-care OR Medicaid OR MMC OR MCO OR coordinated-care OR coordinated-health-care OR HMO OR health-maintenance-organization* OR PPO OR Preferred-Provider-Organizations OR POS OR point-of-service-plan* OR EPO OR exclusive-provider-organization*):ti,ab,kw	8670
#12	#9 OR #10 OR #11	8672
#13	MeSH descriptor: [fee-for-service plans]	91
#14	(Fee-for-Service OR fee-for-services OR fees-for-service OR fees-for-services OR FFS):ti,ab,kw	720
#15	#13 OR #14	755
#16	#8 AND #12 AND #16	28

**Table:**

APA Psycinfo		
Number	Search	Results
S1	DE “Foster Care” OR DE “Foster Children” OR DE “Foster Parents”	8193
S2	TI ((child OR children* OR childhood OR kid OR kids OR adolescent OR adolescents OR adolescence OR preteen* OR teenager* OR teen OR teens OR toddler OR toddlers OR preschool* OR school-age* OR girl OR girls OR girlhood OR boy OR boys OR boyhood OR youth OR pediatric OR pediatrics OR Foster-care OR state-care OR foster-parent* OR foster-mother* OR foster-father* OR kinship-care OR kinship-provider* OR guardian OR guardians OR guardianship OR DCSF)) OR AB ((child OR children* OR childhood OR kid OR kids OR adolescent OR adolescents OR adolescence OR preteen* OR teenager* OR teen OR teens OR toddler OR toddlers OR preschool* OR school-age* OR girl OR girls OR girlhood OR boy OR boys OR boyhood OR youth OR pediatric OR pediatrics OR Foster-care OR state-care OR foster-parent* OR foster-mother* OR foster-father* OR kinship-care OR kinship-provider* OR guardian OR guardians OR guardianship OR DCSF))	1,033,282
S3	S1 OR S2	1,033,611
S4	DE “Managed Care” OR DE “Health Maintenance Organizations”	5137
S5	TI (Managed-care OR Medicaid OR MMC OR MCO OR coordinated-care OR coordinated-health-care OR HMO OR health-maintenance-organization* OR PPO OR Preferred-Provider-Organizations OR POS OR point-of-service-plan* OR EPO OR exclusive-provider-organization) OR AB (Managed-care OR Medicaid OR MMC OR MCO OR coordinated-care OR coordinated-health-care OR HMO OR health-maintenance-organization* OR PPO OR Preferred-Provider-Organizations OR POS OR point-of-service-plan* OR EPO OR exclusive-provider-organization)	15,855
S6	S4 OR S5	17,719
S7	TI (Fee-for-Service OR fee-for-services OR fees-for-service OR fees-for-services OR FFS) OR AB (Fee-for-Service OR fee-for-services OR fees-for-service OR fees-for-services OR FFS)	1312
S8	S3 AND S6 AND S7	71

**Table:**

Social services abstracts (ProQuest)		
Number	Search	Result
S1	MAINSUBJECT.EXACT(“Foster children”)	3052
S2	noft(child OR children* OR childhood OR kid OR kids OR adolescent OR adolescents OR adolescence OR preteen* OR teenager* OR teen OR teens OR toddler OR toddlers OR preschool* OR school-age* OR girl OR girls OR girlhood OR boy OR boys OR boyhood OR youth OR pediatric OR pediatrics OR Foster-care OR state-care OR foster-parent* OR foster-mother* OR foster-father* OR kinship-care OR kinship-provider* OR guardian OR guardians OR guardianship OR DCSF)	160,898
S3	[S1] OR [S2]	160,898
S4	MAINSUBJECT.EXACT.EXPLODE(“Managed care”)	525
S5	noft(Managed-care OR Medicaid OR MMC OR MCO OR coordinated-care OR coordinated-health-care OR HMO OR health-maintenance-organization* OR PPO OR Preferred-Provider-Organizations OR POS OR point-of-service-plan* OR EPO OR exclusive-provider-organization)	6615
S6	[S4] OR [S5]	6615
S7	noft(Fee-for-Service OR fee-for-services OR fees-for-service OR fees-for-services OR FFS)	417
S8	[S3] AND [S6] AND [S7]	31

**Table:**

Web of Science Core Collection (Clarivate)		
Number	Search	Results
#1	TS = (child OR children* OR childhood OR kid OR kids OR adolescent OR adolescents OR adolescence OR preteen* OR teenager* OR teen OR teens OR toddler OR toddlers OR preschool* OR school-age* OR girl OR girls OR girlhood OR boy OR boys OR boyhood OR youth OR pediatric OR pediatrics OR Foster-care OR state-care OR foster-parent* OR foster-mother* OR foster-father* OR kinship-care OR kinship-provider* OR guardian OR guardians OR guardianship OR DCSF)	3,133,471
#2	TS = (Managed-care OR Medicaid OR MMC OR MCO OR coordinated-care OR coordinated-health-care OR HMO OR health-maintenance-organization* OR PPO OR Preferred-Provider-Organizations OR POS OR point-of-service-plan* OR EPO OR exclusive-provider-organization)	119,831
#3	TS = (Fee-for-Service OR fee-for-services OR fees-for-service OR fees-for-services OR FFS)	11,109
#4	#3 AND #2 AND #1	246
